# CHA_2_DS_2_-VASc score as a prognostic indicator in patients with atrial fibrillation undergoing coronary stenting

**DOI:** 10.55730/1300-0144.5413

**Published:** 2022-04-16

**Authors:** Jian-Yong ZHENG, Dong-Tao LI, Yi-Gang QIU, Yi-Xiong HUANG, Zheng-Ming XU, Li ZHAO, Yu CHEN, Yi CAO, Yi-Da TANG, Cheng-Jun GUO, Zhi-Min MA, Yong-Quan WU, Yan JIAO, Tian-Chang LI

**Affiliations:** 1 Department of Cardiology, Division of Cardiology and Cardiovascular Surgery, Sixth Medical Center of PLA General Hospital, Beijing, China; 2 Department of Cardiology, Fuwai Hospital, Chinese Academy of Medical Sciences, Beijing, China; 3 Department of Cardiology, Beijing Anzhen Hospital, Capital Medical University, Beijing, China; 4 Department of Cardiology, Beijing Tongren Hospital, Capital Medical University, Beijing, China; 5 Department of Cardiology, Beijing Friendship Hospital, Capital Medical University, Beijing, China; 6 Catheter Lab for Cardiovascular and Neurological Intervention, Binzhou Medical University Hospital, Binzhou, Shandong Province, China

**Keywords:** Coronary atherosclerotic disease, atrial fibrillation, percutaneous coronary intervention, CHA_2_DS_2_-VASc score, prognosis

## Abstract

**Background/aim:**

Patients with atrial fibrillation (AF) and coronary stenting had a poor prognosis. This study aimed to assess the accuracy of CHA_2_DS_2_-VASc score for predicting and grading adverse clinical outcomes in this population.

**Materials and methods:**

We reviewed the clinical data of all patients with previously documented nonvalvular AF who underwent coronary stenting between January 2010 and June 2015 in 12 hospitals of Beijing, China. The study population was divided into three groups: 1) Low CHA_2_DS_2_-VASc score, ≦ 2 points, 2) Intermediate score, 3–4 points, and 3) High score, ≧ 5 points. Major adverse cardiac/cerebrovascular events (MACCE) were defined as a composite of all-cause death, nonfatal myocardial infarction, repeat revascularization and ischemic stroke/systemic thromboembolism (IS/SE).

**Results:**

A total of 2394 patients (men: 72.3% vs. women: 27.7%, median age: 67 years) were included, with the CHA_2_DS_2_-VASc score of 3.6 ± 1.6. The median follow-up duration was 36.2 months. All-cause mortality increased 3 folds from the low score (4.8%) to the high score group (15.8%). The high score group had more IS/SE (7.4%) and MACCE (26.3%). The CHA_2_DS_2_-VASc score ≧ 5 points was independently associated with all-cause death (hazard ratio [HR]: 2.303, 95% confidence interval [CI]: 1.492–3.555), IS/SE (HR: 4.169, 95% CI: 2.216–7.845) and MACCE (HR: 1.468, 95% CI: 1.113–1.936) on multivariate Cox proportional hazards regression. The area under the receiver operating characteristic curve of the CHA_2_DS_2_-VASc score was 0.644 (95% CI: 0.624–0.663) for all-cause death, 0.647 (95% CI: 0.627–0.666) for IS/SE, and 0.592 (95% CI: 0.572–0.611) for MACCE.

**Conclusion:**

CHA_2_DS_2_-VASc score was a reliable prognostic indicator in patients with AF and coronary stenting.

## 1. Introduction

Nowadays, percutaneous coronary intervention (PCI) with stenting remains the most effective treatment to relieve symptoms in patients with coronary atherosclerotic disease (CAD) and improve prognosis in the acute setting. Approximately 4.5%–12.3% of patients undergoing PCI had atrial fibrillation (AF) [1–6]. In the past decade, cardiologists have focused their attention on this special population who showed an increased thromboembolic risk and worse prognosis compared with those without AF [1–4].

The CHA_2_DS_2_-VASc score is the most widely used scoring system to assess the risk of ischemic stroke and systemic thromboembolism (IS/SE) in patients with AF. In addition, some major components of the CHA_2_DS_2_-VASc score are also risk factors that predispose to CAD. Recent studies also suggested its potential role in predicting adverse clinical outcomes in patients with CAD in the absence of AF, including those undergoing PCI [7]. Thus, we speculated the CHA_2_DS_2_-VASc score would be a reliable algorithm for grading the risk of death and systemic thromboembolism in patients with AF who underwent PCI. In this multicenter observational study, we aimed to assess the accuracy of CHA_2_DS_2_-VASc score for predicting and grading adverse clinical outcomes in a Chinese cohort with AF and coronary stenting.

## 2. Materials and methods

This cross-sectional study included all patients with CAD and previously documented AF who underwent PCI between January 2010 and June 2015 in 12 hospitals of Beijing, China. The exclusion criteria included: 1) Patients with valvular AF (severe mitral stenosis and/or insufficiency); 2) those who had PCI but without stent implantation. The CHA_2_DS_2_-VASc score was calculated according to the interpretations in recent guidelines [8]. We arbitrarily divided the study population into three groups based on their scores: 1) Low score, ≦ 2 points, 2) Intermediate score, 3–4 points, and 3) High score, ≧ 5 points.

Heart failure (HF) was referred to recent decompensated episode irrespective of left ventricular ejection fraction, or the presence of moderate-to-severe left ventricular systolic impairment on cardiac imaging even if asymptomatic [8]. We calculated creatinine clearance with the Cockcroft-Gault equation. Chronic kidney disease (CKD) was referred to moderate-to-severe renal dysfunction based on calculated creatinine clearance < 60 mL/min. We referred antithrombotic strategy as the one that was initiated following PCI and extended for at least one month after discharge.

All patients were followed up in the outpatient departments or by telephone. We defined the major adverse cardiac/cerebrovascular events (MACCE) as a composite endpoint of all-cause death, nonfatal myocardial infarction, repeat revascularization, and IS/SE. The study was conducted in accordance with the ethical guidelines in the Declaration of Helsinki. The ethics committee of the primary investigating institution approved the study protocol (approval number: HZKY-PJ-2021-23). Informed consents were obtained from all participants in the study.

The sample size was calculated with G*Power software version 3.1. We considered that an appropriate sample size of 1545 would be adequate to demonstrate significant differences in mortality and MACCE among the three score groups, based on an α error probability of 0.05, power of 0.95, and effect size w of 0.1. Statistical analysis was performed with IBM SPSS Statistics version 20.0 and MedCalc version 19.7. The correlation of the CHA_2_DS_2_-VASc score with GRACE score was assessed with Pearson or Spearman correlation coefficient, according to the normality of distribution. We compared basic clinical characteristics and mid-term adverse clinical outcomes between low, intermediate and high score groups. Normally distributed continuous variables were presented as mean and standard deviation, and compared with the one-way ANOVA (LSD and S-N-K for post hoc multiple comparisons). Nonnormally distributed continuous variables were expressed as median (interquartile range), and compared with the Kruskal-Wallis H test (with missing group for post hoc intergroup comparisons). Categorical variables were reported as numbers (percentage), and compared with Pearson chi-square test. In order to further assess the independent effect of the CHA_2_DS_2_-VASc score on adverse clinical outcomes, we performed multivariate Cox proportional hazards regression to correct for the baseline imbalance and potential effect of other covariates. The performance of covariates was measured with a hazard ratio (HR) with its 95% confidence interval (CI). In addition, a receiver operating characteristic (ROC) curve was generated for the CHA_2_DS_2_-VASc score, with the area under the curve (AUC) representing the predictive performances of the CHA_2_DS_2_-VASc score for all-cause death, systemic thromboembolism, and MACCE. A two-sided P-value lower than 0.05 indicated statistical significance. In addition, a Bonferroni adjustment was made for the P-value, and a corrected P-value less than 0.017 was adopted for intergroup comparisons.

## 3. Results

**Figure 1:**
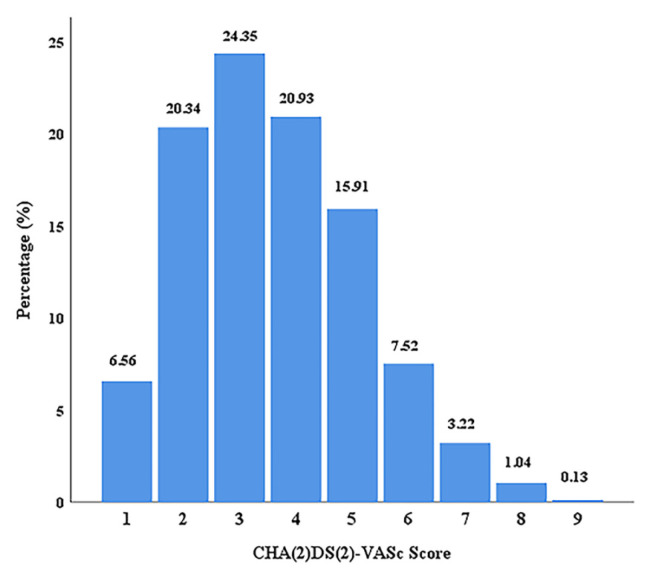
The distribution of the CHA_2_DS_2_-VASc score in the study population.

A total of 2394 (men: 72.3% vs. women: 27.7%, median age: 67 years) out of the 2511 patients who were eligible for the inclusion criteria had complete follow-up data, and constituted the study population. The CHA_2_DS_2_-VASc score was 3.6 ± 1.6 at baseline. The distribution of the CHA_2_DS_2_-VASc score is illustrated in Figure 1.

Table 1 showed the clinical characteristics according to the CHA_2_DS_2_-VASc score. In addition to a greater prevalence of risk factors incorporated in the CHA_2_DS_2_-VASc score (older age, female sex, hypertension, diabetes, HF and previous ischemic stroke), patients with higher scores were also more likely to have anemia and CKD compared with those with lower scores. There were no significant differences with regard to multivessel disease, target vessel distribution, number of stents implanted and antithrombotic strategy.

Dual antiplatelet therapy was the dominant antithrombotic strategy following PCI in each group. Our cohort had a high usage rate of β receptor blockers (77.1%), angiotensin converting enzyme inhibitors/angiotensin receptor blockers (60.2%) and statins (94.3%). Patients in the low score group were less treated with angiotensin converting enzyme inhibitors/angiotensin receptor blockers. Statins were significantly less commonly used with increasing CHA_2_DS_2_-VASc score, although the difference was marginal.

**Figure 2:**
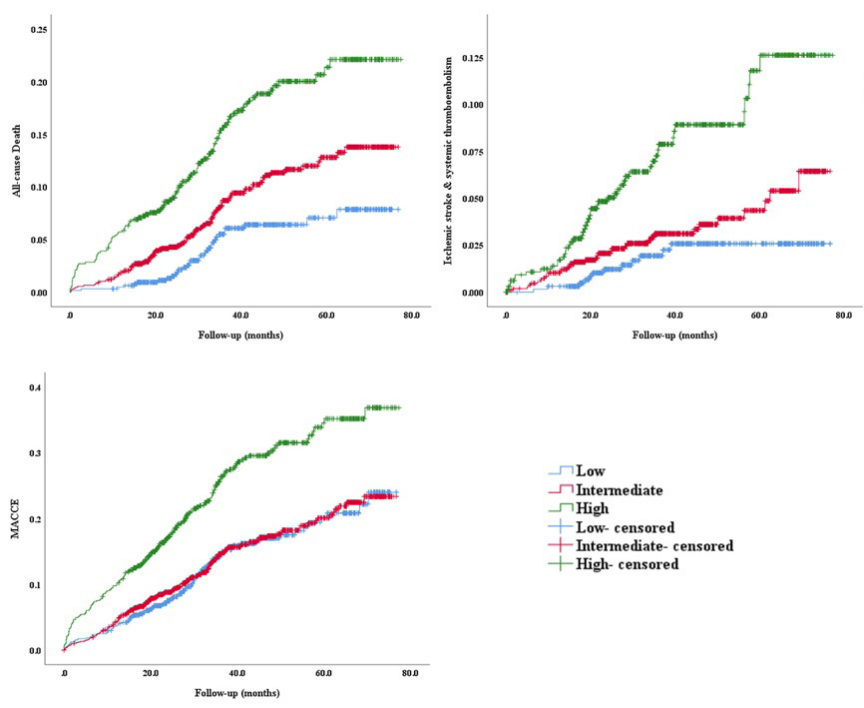
The mid-term all-cause mortality, incidence of ischemic stroke & systemic thromboembolism and MACCE following percutaneous coronary intervention according to the CHA_2_DS_2_-VASc score. MACCE: major adverse cardiac/cerebrovascular events.

The median follow-up duration was 36.2 months. Table 2 and Figure 2 showed the mid-term clinical outcomes following PCI according to the CHA_2_DS_2_-VASc score. All-cause mortality increased more than 3 folds from the low score (4.8%) to the high score group (15.8%). The low score group had the highest repeat revascularization rate (7.9%), while the high score group had more IS/SE (7.4%) and MACCE (26.3%) during follow-up.

**Figure 3:**
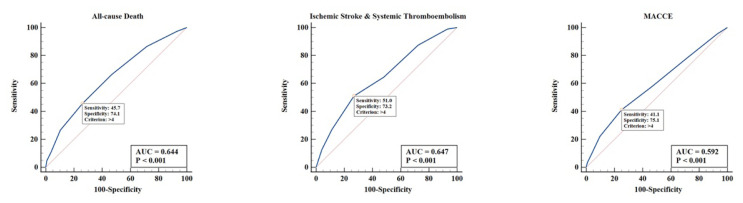
The receiver operating characteristic curve of the CHA_2_DS_2_-VASc score for the prediction of all-cause death, ischemic stroke & systemic thromboembolism and MACCE. AUC: area under the curve; MACCE: major adverse cardiac/cerebrovascular events.

The CHA_2_DS_2_-VASc score ≧ 5 points was independently associated with all-cause death (HR: 2.303, 95% CI: 1.492–3.555), IS/SE (HR: 4.169, 95% CI: 2.216–7.845) and MACCE (HR: 1.468, 95% CI: 1.113–1.936) on multivariate Cox proportional hazards regression. Other independent predictors in the regression models for adverse clinical outcomes are demonstrated in Table 3. The ROC curve analysis is illustrated in Figure 3. The AUC of the CHA_2_DS_2_-VASc score was 0.644 (95% CI: 0.624–0.663) for all-cause death, 0.647 (95% CI: 0.627–0.666) for IS/SE, and 0.592 (95% CI: 0.572–0.611) for MACCE. The CHA_2_DS_2_-VASc score > 4 points had 1) a sensitivity of 45.7%, specificity of 74.1%, positive predictive value (PPV) of 15.8% and negative predictive value (NPV) of 92.8% for all-cause death; 2) a sensitivity of 51.0%, specificity of 73.2%, PPV of 7.4% and NPV of 97.3% for IS/SE; 3) a sensitivity of 41.1%, specificity of 75.1%, PPV of 26.3% and NPV of 85.5% for MACCE. In addition, the CHA_2_DS_2_-VASc score had a positive correlation with the GRACE score (median: 116, interquartile range: 101–135; Spearman coefficient r = 0.366, p < 0.001).

## 4. Discussion

In our cohort with AF and coronary stenting, incremental increase in CHA_2_DS_2_-VASc score was closely associated with adverse clinical outcomes during a median follow-up period of 36 months. With multivariate Cox proportional hazards regression, CHA_2_DS_2_-VASc score ≧ 5 points was an independent predictor for all-cause death, IS/SE, and MACCE.

In this observational study, we adopted the latest criteria of the components in the CHA_2_DS_2_-VASc score. The CHA_2_DS_2_-VASc score has long been used to assess the risk of IS/SE in patients with AF. Since the first introduction of the risk score, the interpretation on the meaning of some covariates has evolved. For example, “C” initially stood for congestive HF with reduced left ventricular ejection fraction (HFrEF). However, AF occurred with progressive HF with preserved ejection fraction (HFpEF) and further aggravated heart dysfunction. According to the recent European guidelines for the diagnosis and management of AF, congestive HF denotes recent decompensated episode irrespective of ejection fraction (thus incorporating HFrEF [ejection fraction < 45%] or HFpEF), or the presence of moderate-severe left ventricular systolic impairment on cardiac imaging even if asymptomatic [8]. In addition, the initial definition of “vascular disease” only incorporated previous myocardial infarction, peripheral artery disease, or aortic plaque. However, subsequent studies suggested that angiographically significant CAD was also an independent risk factor for ischemic stroke among AF patients [9]. Therefore, we adopted the “vascular disease” criterion in recent AF guidelines which advocated inclusion of CAD in the calculation of the CHA_2_DS_2_-VASc score [8].

In addition to its clinical application in the field of AF, several studies also investigated the prognostic value of the CHA_2_DS_2_-VASc score in patients with CAD in the absence of AF. In a Chinese cohort of 3745 patients with acute coronary syndrome who underwent PCI, higher CHA_2_DS_2_-VASc score was independently associated with an increased risk of MACCE during a median follow-up period of 33 months [7]. In a recent retrospective analysis on 906 patients with non-ST segment elevation myocardial infarction, the CHA_2_DS_2_-VASc score had a significant positive correlation with the SYNTAX score, and was independently associated with in-hospital mortality [10]. These findings also help to explain why increased CHA_2_DS_2_-VASc score indicated adverse clinical outcomes in patients with AF and coronary stenting as shown in our study.

In our Chinese cohort, a higher score (≧ 5 points) was associated with increased mid-term mortality and incidence of IS/SE and MACCE. Similarly, a retrospective analysis of the US National Inpatient Sample Database demonstrated that an incremental increase in the CHA_2_DS_2_-VASc score was independently associated with in-hospital death, stroke and adverse periprocedural events following PCI in patients presenting with acute coronary syndrome and concomitant AF [11]. In addition, increasing CHA_2_DS_2_-VASc score was associated with increased risk of stroke and higher mortality in an Australian registry of 564 AF patients undergoing PCI [12]. The association reflected the fact that the major components in the CHA_2_DS_2_-VASc score were common risk factors and prognostic indicators for both CAD and AF [6,8,13,14]. In addition, our study showed a good correlation of the CHA2DS2-VASc score with the GRACE score, which has long been demonstrated to predict adverse clinical outcomes reliably in acute coronary syndrome. The predictive value of the CHA_2_DS_2_-VASc score for adverse clinical outcomes as measured with ROC analysis was comparable between our study and a French analysis. The AUC of the CHA_2_DS_2_-VASc score was 0.644 (95% CI: 0.624–0.663) versus 0.603 (95% CI: 0.566–0.640) for all-cause death, 0.647 (95% CI: 0.627–0.666) versus 0.586 (95% CI: 0.549–0.622) for IS/SE, and 0.592 (95% CI: 0.572–0.611) versus 0.543 (95% CI: 0.507–0.580) for MACCE [15].

As a well-established scoring system, the CHA_2_DS_2_-VASc score appeared to be a promising indicator for clinical outcomes in this population. It was easy to be calculated with known clinical risk factors even during first medical contact and precluded the acquisition of complex clinical data not readily accessible and complicated calculation. The convenience for clinical application allowed more intensive medical and more active interventional treatment for high-risk patients. However, whether these measures would improve clinical outcomes remains to be determined.

In the multivariate analysis, we also identified other covariates that were not incorporated into the CHA_2_DS_2_-VASc score. In consistency with the AFCAS registry, CKD was independently associated with all-cause mortality and MACCE in our cohort [16]. Firstly, patients with CKD often had more advanced atherosclerosis with more diffuse and calcified lesions. Besides, CKD disorders the thrombotic process, and complicates the metabolism of cardiovascular medications [17]. In addition, CKD promotes inflammation and activates neurohormonal signaling pathways. All these pathophysiologic changes had a detrimental effect on clinical outcomes. Another prominent risk factor for all-cause death and MACCE was history of myocardial infarction (either previous or current), while statin use was the most protective treatment. Aside from the CHA_2_DS_2_-VASc score, intracranial hemorrhage was the other risk factor associated with IS/SE during follow-up. Similarly, intracranial hemorrhage had also been shown to be associated with subsequent IS/SE in a large-scale Danish AF registry [18].

Our cohort had a high prevalence of β receptor blocker, angiotensin converting enzyme inhibitor/angiotensin receptor blocker and statin use. It was worthy of note that patients with intermediate scores were more likely to be treated with β receptor blockers, perhaps because patients with higher scores usually had an older age with lower heart rates. Patients with low scores were less treated with angiotensin-converting enzyme inhibitors/angiotensin receptor blockers due to a less prevalence of hypertension in this group.

A large number of our study population had an intermediate-to-high thrombotic risk according to the CHA_2_DS_2_-VASc score. However, only a small minority (5.6%) was given anticoagulation. This finding was in contrast with current guidelines and practice, in which Clopidogrel in combination with warfarin or a novel non-Vitamin K oral anticoagulant was the antithrombotic strategy of choice in patients with AF who underwent PCI [8, 19–23]. However, our patients received PCI procedures before the current guidelines and expert consensus were published. The inadequate anticoagulation in this study was speculated to result from the concern from many Chinese cardiologists on the bleeding risk following PCI when oral anticoagulants was combined with antiplatelet agents. Actually, warfarin was inadequately used even in the general AF population in China, mainly due to patient unwillingness to receive regular INR monitoring and concern of bleeding [24]. Moreover, novel non-Vitamin K oral anticoagulants were not covered by the local medical insurances, and thus not widely used during the study period.

There are some limitations to this study. The clinical data was derived from different medical centers. As with all multicenter retrospective studies, there was no audit of data quality and precision. The cause of death was missing, unclear or inaccurate for a large number of patients in the cohort, and therefore we could not discriminate cardiac from noncardiac death. We did not calculate the SYNTAX score, which showed a positive correlation with the CHA2DS2-VASc score and was independently associated with mortality, as evidenced in some recent studies [10].

In conclusion, patients with AF and high CHA_2_DS_2_-VASc score who underwent PCI with stenting had a poor mid-term prognosis. The CHA_2_DS_2_-VASc score was a reliable index for risk stratification of adverse clinical outcomes following PCI, with ≧ 5 points independently associated with all-cause death, IS/SE, and MACCE.

## Figures and Tables

**Table 1:** Clinical characteristics according to the CHA_2_DS_2_-VASc score.

	Low (n = 644)	Intermediate (n = 1084)	High (n = 666)	P value
Age (years)	59.0 (54.0–63.0)*	68.0 (62.0–73.0)*	75.0 (69.0–78.0)*	<0.001
Female, n (%)	19 (3.0)*	270 (24.9)*	373 (56.0)*	<0.001
Hypertension, n (%)	289 (44.9)*	862 (79.5)*	611 (91.7)*	<0.001
Diabetes, n (%)	40 (6.2)*	333 (30.7)*	357 (53.6)*	<0.001
Current smoker, n (%)	404 (62.7)*	474 (43.7)*	198 (29.7)*	<0.001
Previous MI, n (%)	16 (2.5)	29 (2.7)	27 (4.1)	0.172
Previous PCI, n (%)	103 (16.0)*	199 (18.4)	150 (22.5)*	0.009
Previous CABG, n (%)	13 (2.0)	43 (4.0)	21 (3.2)	0.085
Previous ischemic stroke, n (%)	0 (0)*	65 (6.0)*	287 (43.1)*	<0.001
Previous major bleeding, n (%)	10 (1.6)	25 (2.3)	22 (3.3)	0.113
Previous intracranial hemorrhage, n (%)	3 (0.5)	4 (0.4)	6 (0.9)	0.323
Previous gastrointestinal bleeding, n (%)	4 (0.6)	1 (0.1)	5 (0.8)	0.075
Anemia, n (%)	30 (4.7)*	165 (15.2)*	187 (28.1)*	<0.001
CKD, n (%)	18 (2.8)*	242 (22.3)*	283 (42.5)*	<0.001
STEMI, n (%)	101 (15.7)	140 (12.9)	113 (17.0)	0.051
Persistent AF, n (%)	120 (18.6)	198 (18.3)	145 (21.8)	0.171
Heart failure, n (%)	42 (6.5)*	186 (17.2)*	216 (32.4)*	<0.001
Target vessel (>75% stenosis)				
LM, n (%)	15 (2.3)	29 (2.7)	9 (1.4)	0.183
LAD, n (%)	341 (53.0)	611 (56.4)	374 (56.2)	0.345
LCX, n (%)	168 (26.1)	301(27.8)	179 (26.9)	0.743
RCA, n (%)	255 (39.6)	412 (38.0)	257 (38.6)	0.806
RI, n (%)	3 (0.5)	4 (0.4)	2 (0.3)	0.886
SVG, n (%)	0 (0)	3 (0.3)	0 (0)	0.163
Multivessel PCI, n (%)	130 (20.2)	239 (22.0)	144 (21.6)	0.653
Number of stents	1.0 (1.0–2.0)	2.0 (1.0–2.0)	2.0 (1.0–2.0)	0.841
Antithrombotic agents				
Triple therapy, n (%)	22 (3.4)	56 (5.2)	31 (4.7)	0.238
Dual antiplatelets, n (%)	614 (95.3)	1021 (94.2)	624 (93.7)	0.410
One antiplatelet plus one oral anticoagulant, n (%)	8 (1.2)	7 (0.6)	11 (1.7)	0.130
Oral anticoagulants, n (%)	30 (4.7)	63 (5.8)	42 (6.3)	0.410
β receptor blockers, n (%)	492 (76.4)	859 (79.2)	495 (74.3)	0.052
ACEI/ARB, n (%)	332 (51.6)*#	696 (64.2)*	414 (62.2)#	<0.001
Statins, n (%)	623 (96.7)*	1024 (94.5)	610 (91.6)*	<0.001
PPI, n (%)	140 (21.7)	273 (25.2)	176 (26.4)	0.120

ACEI: angiotensin converting enzyme inhibitor; AF: atrial fibrillation; ARB: angiotensin receptor blocker; CABG: coronary artery bypass grafting surgery; CKD: chronic kidney disease; LAD: left anterior descending coronary artery; LCX: left circumflex coronary artery; LM: left main coronary artery; MI: myocardial infarction, PCI: percutaneous coronary intervention; PPI: proton-pump inhibitor; RCA: right coronary artery; RI: ramus intermedius; STEMI: ST segment elevation myocardial infarction; SVG: saphenous vein graft

* and # indicate significant difference (adjusted P value < 0.017) between groups.

**Table 2:** Clinical outcomes following PCI according to CHA_2_DS_2_-VASc score.

	Low (n = 644)	Intermediate (n = 1084)	High (n = 666)	P-value
All-cause death, n (%)	31 (4.8) *	94 (8.7) *	105 (15.8) *	<0.001
Myocardial infarction, n (%)	3 (0.5)	7 (0.6)	12 (1.8)	0.018
Repeat revascularization, n (%)	51 (7.9) *#	34 (3.1) *	28 (4.2) #	<0.001
Ischemic stroke & systemic thromboembolism, n (%)	12 (1.9) *	35 (3.2) #	49 (7.4) *#	<0.001
MACCE, n (%)	91 (14.1) *	160 (14.8) #	175 (26.3) *#	<0.001
Major bleeding, n (%)	17 (2.6)	35 (3.2)	20 (3.0)	0.786

MACCE: major adverse cardiovascular/cerebral events; PCI: percutaneous coronary intervention

* and # indicate significant difference (adjusted p value < 0.017) between groups.

**Table 3:** Predictive performance of the CHA_2_DS_2_-VASc score for adverse clinical outcomes on multivariate proportional hazards regression.

Items	Odds ratio (95% Confidence interval)	P-value
**All-cause death**		
CHA2DS2-VASc score (referenced by ≦ 2 points)		
3–4 points	1.527 (1.004–2.324)	0.048
≧ 5 points	2.303 (1.492–3.555)	<0.001
Previous MI	2.280 (1.321–3.934)	0.003
CKD	2.085 (1.572–2.766)	<0.001
STEMI at presentation	1.958 (1.452–2.640)	<0.001
ACEI/ARB	0.745 (0.572–0.970)	0.029
Statins	0.414 (0.283–0.604)	<0.001
**Ischemic stroke & systemic thromboembolism**		
CHA2DS2-VASc score (referenced by ≦ 2 points)		
3–4 points	1.785 (0.926–3.438)	0.083
≧ 5 points	4.169 (2.216–7.845)	<0.001
Previous intracranial hemorrhage	5.642 (1.777–17.914)	0.003
**MACCE**		
CHA2DS2-VASc score (referenced by ≦ 2 points)		
3–4 points	0.895 (0.686–1.168)	0.415
≧ 5 points	1.468 (1.113–1.936)	0.007
Previous MI	1.915 (1.256–2.919)	0.003
Previous PCI	1.261 (1.000–1.590)	0.050
CKD	1.711 (1.378–2.123)	<0.001
STEMI at presentation	1.764 (1.403–2.218)	<0.001
Statins	0.448 (0.330–0.607)	<0.001

ACEI: angiotensin converting enzyme inhibitor; ARB: angiotensin receptor blocker; CKD: chronic kidney disease; MI: myocardial infarction; PCI: percutaneous coronary intervention; STEMI: ST segment elevation myocardial infarction
